# Concurrent Palmar Lunate Dislocation and Posterior Elbow Dislocation With a Distal Radius Fracture: A Rare Case Report

**DOI:** 10.7759/cureus.52609

**Published:** 2024-01-20

**Authors:** Ousama Jelti, Abdelilah Barzouq, Oussama El Alaoui, Najib Abdeljaouad, Hicham Yacoubi

**Affiliations:** 1 Department of Orthopedics and Traumatology, Faculty of Medicine and Pharmacy, Mohammed VI University Hospital, Mohammed First University, Oujda, MAR; 2 Department of Orthopedics and Traumatology, Faculty of Medicine and Pharmacy, Centre Hospitalier Universitaire (CHU) Mohammed VI, Oujda, MAR; 3 Department of Orthopedics and Traumatology, Faculty of Medicine and Pharmacy, Centre Hospitalier Universitaire (CHU) Mohammed VI, Mohammed First University, Oujda, MAR

**Keywords:** surgery, floating forearm, wrist injuries, elbow joint, dislocation

## Abstract

We present an unusual case involving the simultaneous dislocation of the trans-scapho-retro-lunate in the right wrist and a posterolateral dislocation in the right elbow joint with a distal radius fracture in a 23-year-old male with no notable medical history. These injuries occurred when he fell from a height of around 12 meters onto his outstretched right hand. The patient presented to the hospital in a normal upper limb trauma position with no discomfort to critical functions. Predominant symptoms at the emergency department were discomfort in the right wrist, hand, and ipsilateral elbow, as well as the entire upper limb functional impairment. Both the wrist and the elbow seemed distorted on examination, with considerable edema and loss of bone landmarks. Passive mobilization was hampered by pain, but peripheral pulses were detectable. The radial, ulnar, and median nerves' autonomous sensory-motor domains were intact, with a cutaneous opening classified as stage 2 by the Cauchoix-Duparc classification. The elbow dislocation was successfully treated using a closed reduction method. External manipulation was employed to reduce the trans-scaphoid perilunate dislocation, which was subsequently stabilized through percutaneous screw fixation of the scaphoid using a triquetrum-lunate pin. Additionally, a styloid pin was utilized to address and manage a distal radius fracture, followed by the implementation of a radiometacarpal external fixator. After one year and three months, the patient reported no pain in his elbow and minimal wrist discomfort during heavy lifting.

## Introduction

The rare co-occurrence of traumatic perilunate and elbow dislocation is only rarely observed. The clinical situation in which unilateral proximal and distal forearm dislocation is seen is referred to as bipolar forearm dislocation or floating forearm, and only twelve cases of this type have been reported in the literature [1-5]. Due to its occurrence following severe trauma, the patient may present with lesions of other systems. Limitations of joint movement, deformity and pain are usually present. In the absence of careful physical examination, perilunate dislocation may go undetected. In most cases, fractures and open wounds of the wrist and elbow are observed. The functional prognosis is closely linked to that of the wrist [6]. The authors presented another example of a floating forearm, this time associated with a distal radius fracture, in their study.

## Case presentation

This case involves a 23-year-old man with no notable medical history who fell from a height of around 12 m. The patient presented to the hospital in a normal upper limb trauma position with no discomfort to critical functions. Predominant symptoms at the emergency department were discomfort in the right wrist, hand, and ipsilateral elbow, as well as the entire upper limb functional impairment. Both the wrist and elbow seemed distorted on examination, with considerable edema and loss of bone landmarks. Passive mobilization was hampered by pain, but peripheral pulses were detectable. The radial, ulnar, and median nerves' autonomous sensory-motor domains were intact, with a cutaneous opening classed as stage 2 by the Cauchoix-Duparc classification.

**Figure 1:**
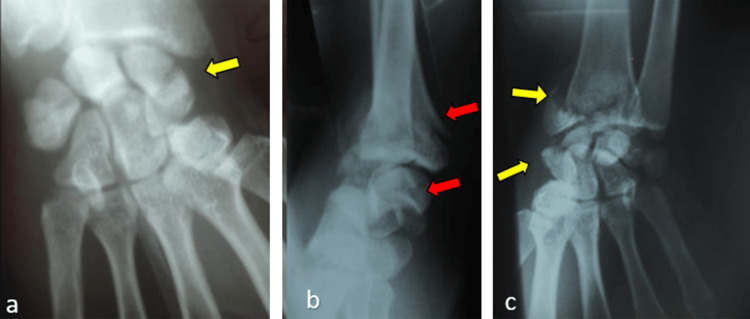
(a) and (c) anteroposterior (AP) radiographs of the wrist showing scaphoid and distal radius fractures (yellow arrows) and (b) profil radiograph showing a trans-scapho retrolunar fracture with a distal radius articular fracture (red arrows).

**Figure 2:**
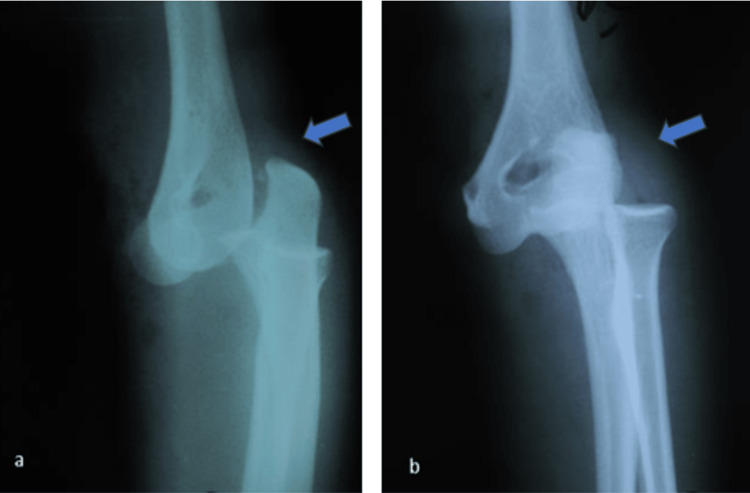
AP (a) and profil (b) radiographs of the elbow showing a posterolateral dislocation (blue arrows).

Urgent radiographic assessment revealed a trans-scaphoid perilunate wrist dislocation, an external cuneiform fracture of the distal radius (Figure 1), and a posterolateral elbow dislocation (Figure 2).

**Figure 3:**
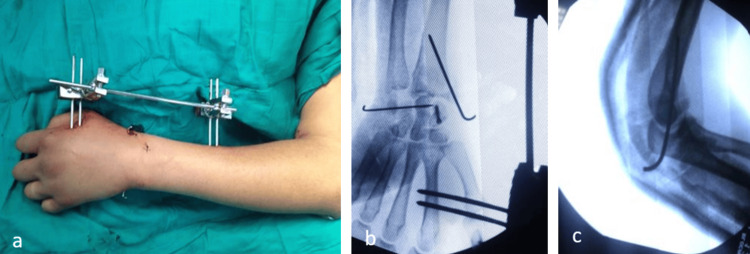
(a) Clinical perioperative image, (b) perioperative fluoroscopic imaging of the wrist, and (c) profil perioperative fluoroscopic picture of the elbow.

This resulted in a floating forearm and a distal radius fracture on the same side. Under general anesthesia, the first step was to reduce the elbow dislocation with external manipulation and kept with a pin, followed by economic debridement of the anterior lesion, which revealed no tendon or vascular-nerve damage. External manipulation was used to reduce the trans-scaphoid perilunate dislocation, which was then stabilized by a percutaneous screw fixation of the scaphoid with a triquetrum-lunate pin. Furthermore, a styloid pin was used to reduce and treat a distal radius fracture, followed by the implantation of a radiometacarpal external fixator (Figure 3).

**Figure 4:**
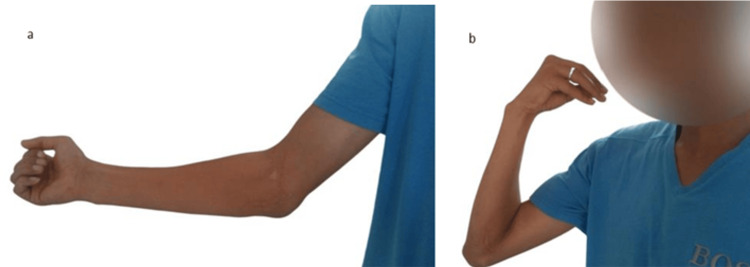
Pictures showing the evolution of elbow extension (a) and flexion (b) after one year and three months.

**Figure 5:**
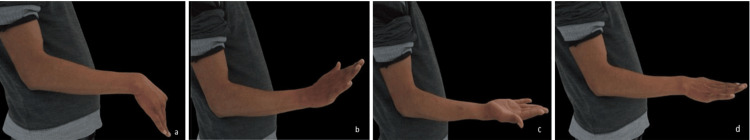
Pictures showing clinical ranges of motion of the wrist after one-year follow-up. (a) Palmar flexion, (b) extension, (c) supination, and (d) pronation.

After radiological confirmation, the patient was discharged on the third postoperative day. At three weeks, the trans-olecranon pin was removed, and at six weeks, the external fixator with the triquetrum-lunate pin was removed. Radiological examinations revealed no secondary displacement. The patient returned to work four and a half months after surgery. The last postoperative follow-up after one year and three months showed a stable and painless elbow with flexion/extension of 135°/- 20° (Figure 4) and a mildly uncomfortable wrist with flexion/extension/prono-supination of 70°/35°/110° (Figure 5).

**Figure 6:**
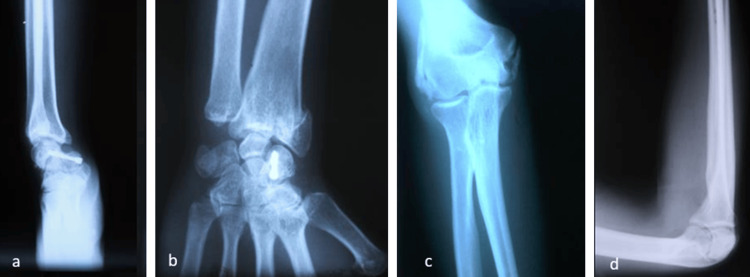
Radiographic images after one year of follow-up. (a-b) AP and profil imaging of the wrist and (c-d) AP and profil radiographs of the elbow.

Control radiographs showed consolidation of wrist fractures with heterotopic classifications in the elbow joint (Figure 6).

## Discussion

The majority of cases with bipolar dislocation of the forearm recorded in the literature are recent; in fact, Masmejean et al. were the first to use this nomenclature to characterize the combination of a genuine posterior dislocation of the elbow and a perilunar dislocation of the wrist [2]. Only twelve occurrences of elbow dislocation associated with perilunar dislocation of the carpus have been recorded in the literature [2-5,7-10]. We did not evaluate Kerr et al.'s observation, in which there is a radial head subluxation rather than a true elbow dislocation [11], nor Rosson's example, in which there is a triple dislocation in the upper limb including the shoulder, elbow, and radio-carpal joint [12]. Moreover, neither Reddy's [13] nor Elloumi's [10] cases, which described a terrible triad injury of the elbow with a peri lunate dislocation of the wrist, were evaluated.

Our observation was unique in that it was the first time in the literature that a floating forearm was accompanied by a partial articular fracture of the distal radius. Almost all cases described in the literature involved young male subjects between the ages of 20 and 35 and sometimes occurred in the context of road accidents. In the case of our injured patient, who fell from a height of around 12 meters, the mechanism indicated by most authors is instinctual palmar support during impact [2-3], communicated by the direct impact of the palm on the ground, to the wrist and elbow in extension, and could also explain the greater frequency of the perilunar dislocations of the carpus. The victim does not have time to prepare for proper reception of the trauma because of the extreme velocity and brutality of the strike.

The diagnosis of this combined injury might be difficult as the trans-scapho-retro-lunar fracture-carpus dislocation may go unreported within the loud clinical picture of an elbow dislocation, especially when polytrauma is present [2-3,5,14]. Diagnostic lag occurs in two out of every five instances, extending from a few days to several weeks, affecting the wrist's functional prognosis [15]. This should urge a thorough and rigorous evaluation of patients to avoid overlooking injuries that may go undiscovered in an emergency and lead to major functional consequences [16]. For the elbow, early orthopedic therapeutic therapy is uncomplicated, with external maneuver reduction followed by immobilization. However, it is surgical and difficult for wrist injuries, especially because it influences the functional prognosis of upper limb apprehension. In this scenario, we underline the significance of immediate reduction and stabilization through pinning. In cases of concomitant scaphoid or distal radius fractures, the intervention is typically intended to include dislocation reduction and scaphoid bone repair by screwing or pinning, followed by capsuloligamentous repair with distal radius pinning utilizing the Kapandji approach.

There is no established treatment strategy for floating forearms. After 10 minutes of manual traction, closed reduction motions of the wrist may be successful [17]. External fixation may help with indirect wrist reduction via ligamentotaxis. However, this technique has yielded unsatisfactory functional outcomes. Thus, polytrauma patients with accompanying ipsilateral extremity fractures, when swelling prevents casting the wrist, or elderly neglected unreduced perilunate dislocations, are the key indications for ligamentotaxis [18]. In all other situations, it appears that early surgical therapy and open reduction are the best options for the wrist. In actuality, a favorable functional outcome is dependent on appropriate carpal bone alignment and the timing of operation [1,17].

However, problems such as superficial infection and ulnar nerve neuropraxia have been described. Patients may tolerate a loss in elbow range of motion as long as it does not interfere with their everyday activities [19]. In our situation, the patient retained a slight limitation in the range of motion of his elbow, which did not affect his daily activities. At the 18-month follow-up, functional outcomes were favorable.

## Conclusions

The unusual simultaneity of elbow and wrist joint dislocation with a distal radius fracture is extremely rare. Given the context frequently associated with polytrauma, we emphasize the importance of early detection of these little-understood injuries, which can easily escape notice in emergencies. In the case of high-energy trauma, neglecting to diagnose elbow and wrist joint dislocations can have disastrous functional consequences. A thorough examination is imperative. Only urgent surgery of the elbow and wrist can lead to favorable results. In our case, the patient regained almost normal function of his right upper limb.
